# Development of a core outcome set for pharmacological interventions in osteoporosis among patients with diabetes mellitus: an international consensus study protocol

**DOI:** 10.3389/fphar.2025.1510968

**Published:** 2025-03-10

**Authors:** Chao Wei, Xiaobin Wang, Zubing Mei, Jing Li

**Affiliations:** ^1^ Department of Spine Surgery, The Second Xiangya Hospital, Central South University, Changsha, China; ^2^ Department of Spine Surgery, The First Affiliated Hospital of Fujian Medical University, Fuzhou, Fujian, China; ^3^ Department of Anorectal Surgery, Shuguang Hospital, Shanghai University of Traditional Chinese Medicine, Shanghai, China; ^4^ Anorectal Disease Institute of Shuguang Hospital, Shanghai, China

**Keywords:** core outcome set, osteoporosis, diabetes mellitus, pharmacological interventions, delphi technique, meta-analysis

## Abstract

**Background:**

Osteoporosis and diabetes mellitus (DM) are both prevalent chronic conditions associated with significant morbidity, particularly in aging populations. Patients with DM are at increased risk of developing osteoporosis due to complex pathophysiological interactions between glucose metabolism and bone health. Although pharmacological interventions have been used to prevent and manage osteoporosis in individuals with DM, variability in reported outcomes across studies hinders evidence synthesis and meta-analyses. A standardized Core Outcome Set (COS) is required to harmonize outcome reporting in clinical trials, improving comparability and clinical relevance. This paper outlines the protocol for developing a COS for pharmacological interventions targeting osteoporosis among patients with DM.

**Methods:**

The development of the COS will follow a five-phase approach. Phase 1 involves a systematic review to identify key outcomes in clinical trials of osteoporosis pharmacotherapy in diabetic populations. Phase 2 consists of a modified Delphi process involving international experts in endocrinology, bone metabolism, and diabetes care, as well as patients and public representatives. This will be followed by Phase 3, where consensus meetings will be held to finalize the essential outcomes for inclusion. Phase 4 will focus on identifying appropriate outcome measurement tools based on a systematic review and additional consensus-building meetings. Finally, Phase 5 will involve dissemination and implementation activities to ensure broad adoption of the COS in future research and clinical trials. Patient and Public Involvement (PPI) will be integrated throughout all phases of the project to ensure the relevance of selected outcomes.

**Conclusion:**

The resulting COS will provide a standardized framework for reporting outcomes in pharmacological intervention studies of osteoporosis in patients with DM. By facilitating meta-analyses and data pooling, this COS will improve the comparability of clinical trials, enhance research efficiency, and reduce outcome reporting bias. Ultimately, the COS will support better clinical decision-making, fostering the development of targeted and effective therapies for osteoporosis in the context of diabetes.

## 1 Introduction

Osteoporosis is a significant global health issue, particularly in older adults, characterized by reduced bone mass and increased susceptibility to fractures. The condition is prevalent among individuals over the age of 50, affecting millions worldwide, and contributing to a high economic burden on healthcare systems (Zanker and Duque, 2019). The skeletal fragility that accompanies osteoporosis is a major cause of morbidity and mortality, particularly due to fractures of the hip, spine, and wrist (Perez et al., 1992). On the other hand, diabetes mellitus (DM) is a chronic metabolic disorder that affects glucose regulation and is increasingly recognized for its adverse effects on bone health (Cipriani et al., 2020). Both type 1 and type 2 diabetes are associated with an elevated risk of osteoporosis and fractures (Wang et al., 2019), but the mechanisms are multifaceted and involve alterations in bone turnover, quality, and microarchitecture. The coexistence of osteoporosis and DM presents unique clinical challenges, as the pathophysiological interactions between glucose metabolism and bone remodeling complicate disease management (Ala et al., 2020). This confluence of conditions increases the complexity of treatment and heightens the urgency for tailored therapeutic strategies.

Pharmacological interventions are a cornerstone in the management of osteoporosis, with the primary goal of improving bone density and reducing the risk of fractures (Qaseem et al., 2023). Several classes of drugs, including bisphosphonates, denosumab, and teriparatide, are widely used in clinical practice. However, their efficacy and safety profiles in patients with DM are not fully understood, partly due to the varying methodologies and outcomes reported in clinical trials. The lack of consistency in outcome measures across studies has hindered the ability to conduct meta-analyses and pool data, limiting the generalizability of findings to this patient population. Consequently, there is a pressing need for the development of a Core Outcome Set (COS) that can standardize outcome reporting in clinical trials evaluating pharmacological interventions for osteoporosis in patients with DM (Bellucci et al., 2021). Establishing a COS will facilitate evidence synthesis, enable better comparability of trial results, and ultimately guide clinical decision-making to improve patient outcomes (Retzer et al., 2020).

The need for a COS is particularly pressing in the context of pharmacological interventions for osteoporosis in patients with DM. Despite numerous clinical trials evaluating the effectiveness of pharmacological interventions (Anagnostis et al., 2018; Paschou et al., 2017; Schwartz, 2017; Mohsin et al., 2019), the lack of standardized outcome measures makes it difficult to compare results across studies. This inconsistency hinders the ability to identify the most effective treatments and to generate high-quality evidence that can inform clinical decision-making. Establishing a COS for this population will provide a structured and standardized approach to outcome reporting, reducing heterogeneity, minimizing bias, and ultimately guiding the identification of the most effective and safe pharmacological treatments for osteoporosis in patients with DM.

This study outlines the protocol for developing a COS for pharmacological intervention trials in osteoporosis among patients with DM, which will serve as a foundation for future research and evidence-based clinical practice. By establishing a standardized set of core outcomes, this initiative aims to improve the quality of research, facilitate evidence synthesis, and support the development of effective treatment strategies for this high-risk population.

## 2 Methods

The development of this COS will follow the established guidelines of the COMET Initiative (Williamson et al., 2017). In accordance with these guidelines, this protocol adheres to the Core Outcome Set-STAndardised Protocol (COS-STAP) statement, ensuring that all methodological aspects are clearly defined and transparent (Kirkham et al., 2019). The primary objective of this project is to establish a COS that can standardize outcome reporting in clinical trials assessing the efficacy and safety of pharmacological interventions for osteoporosis in patients with DM.

### 2.1 Project oversight

This project is coordinated by an international Steering Group comprising experts in endocrinology, diabetes, osteoporosis, and evidence-based medicine, ensuring a broad, multidisciplinary approach. The Steering Group includes members from diverse geographical regions, encompassing North America, Europe, Asia, and other regions with high burdens of osteoporosis and diabetes. This diversity allows for consideration of healthcare system variations, ensuring that the final COS is applicable globally, including in low- and middle-income countries (LMICs). The Steering Group will work closely with international collaborators to ensure that outcomes reflect real-world clinical priorities across different healthcare settings. The Steering Group oversees all phases of the project, ensuring that the COS development process is rigorous, transparent, and aligned with international standards. A smaller Core Group, comprised of 3–4 members from the Steering Group, will be responsible for conducting the hands-on work for each phase of the project, while the entire Steering Group will provide ongoing feedback and guidance.

### 2.2 Scope

The COS is designed to evaluate the efficacy and safety of pharmacological interventions, specifically in the context of osteoporosis treatment in individuals with DM. Given the unique pathophysiological challenges presented by the coexistence of osteoporosis and DM, the COS will prioritize outcomes that are both clinically relevant and capable of capturing the specific therapeutic needs of this population. This COS will be applicable across various clinical settings and will aim to address both primary and secondary outcomes commonly reported in randomized controlled trials (RCTs) related to osteoporosis interventions in patients with DM.

### 2.3 Project phases

**FIGURE 1:**
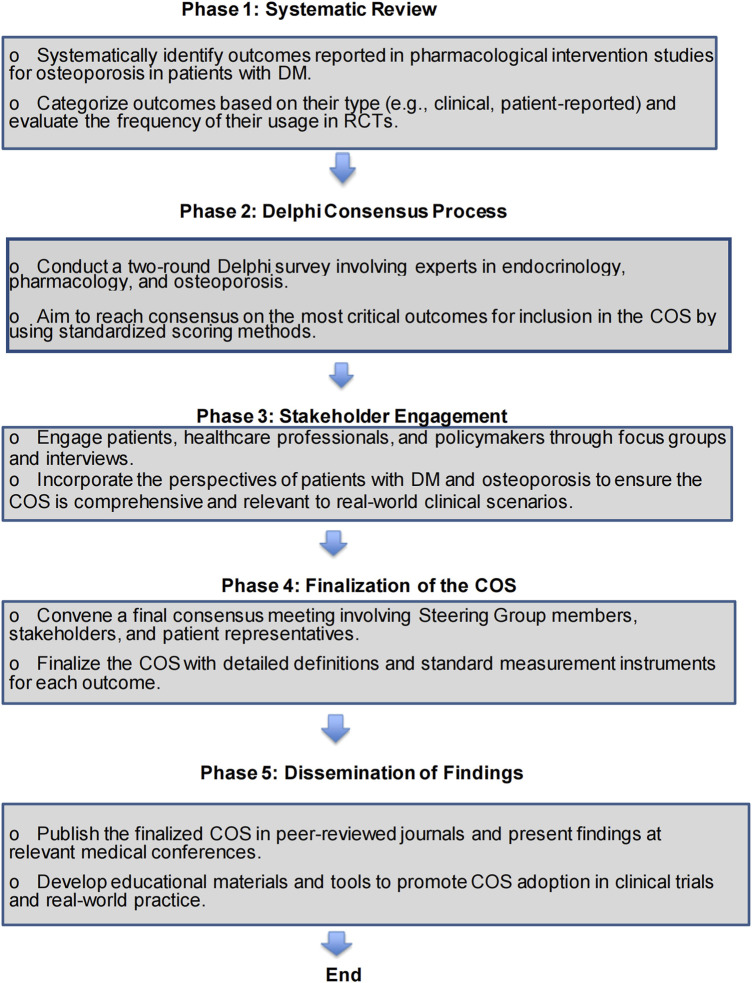
The flow chart of the study process.

The development of this COS will follow five distinct phases, as shown in Figure 1. The phases include a systematic review, a Delphi consensus process, stakeholder engagement, finalization of the COS, and dissemination of the findings (Ingoe et al., 2020). Each phase is designed to ensure a comprehensive and collaborative approach to COS development.

#### 2.3.1 Phase 1: systematic review

A systematic review will be conducted from inception through October 2024 to identify the range of outcomes reported in clinical trials involving pharmacological interventions for osteoporosis in patients with DM. The detailed protocol has been registered in PROSPERO website (International prospective register of systematic reviews), with the registration number of CRD42024576201. The aim of this review was to provide an overview of existing outcome measures and to assess their frequency and methods of evaluation. The inclusion criteria for the systematic review are outlined in Table 1. These trials will be categorized based on their setting (e.g., hospital, community) and outcomes were reported according to their primary and secondary classifications. The results of this systematic review will serve as a foundation for the subsequent phases of the COS development process.

**TABLE 1:** Inclusion criteria for the systematic review.

Criteria	Description
Population	Adults aged 18 years or older diagnosed with both osteoporosis and diabetes mellitus (type 1 or type 2). The studies must specifically identify these patients, and sub-group analysis for patients with DM must be included.
Intervention	Pharmacological treatments aimed at managing osteoporosis, including but not limited to bisphosphonates, denosumab, selective estrogen receptor modulators (SERMs), teriparatide, or other osteoporosis medications. Studies involving combination therapy targeting both osteoporosis and diabetes mellitus outcomes were also considered.
Comparator	Studies comparing pharmacological intervention with placebo, no treatment, or an active comparator (e.g., another osteoporosis drug). Both head-to-head and placebo-controlled trials were included.
Outcomes	Primary outcomes include fracture incidence (vertebral, non-vertebral, hip fractures), changes in bone mineral density (BMD), and adverse effects specific to pharmacological agents. Secondary outcomes include markers of bone turnover, quality of life assessments using validated tools (e.g., EQ-5D, SF-36), incidence of hyperglycemia or hypoglycemia, and patient-reported outcomes (e.g., pain levels, mobility).
Study Type	Randomized controlled trials (RCTs) of any duration. Open-label, double-blind, and crossover studies were included to encompass a broad range of evidence on the intervention’s efficacy and safety.
Setting	Studies conducted in any clinical setting including hospitals, outpatient clinics, or community-based health facilities. Different healthcare system settings were included to ensure broad applicability of outcomes.
Exclusion Criteria	Studies involving non-pharmacological interventions (e.g., exercise, dietary supplements), mixed populations without specific analysis for osteoporosis and diabetes mellitus, and studies not reporting original data (e.g., reviews, meta-analyses).

#### 2.3.2 Phase 2: delphi consensus process

Phase 2 will employ a modified Delphi method, conducted internationally and online in English, to achieve consensus on the core outcomes for pharmacological interventions in osteoporosis among patients with diabetes mellitus (DM). This approach allows for structured communication among experts and stakeholders, facilitating the identification and prioritization of key outcomes. The Delphi survey will be conducted in multiple rounds, each aiming to refine the list of outcomes through a systematic, anonymous process. This iterative approach is designed to reach a consensus on the most relevant outcomes, considering their importance in both clinical trials and real-world settings. Where necessary, the consensus process will also involve stratifying outcomes by various clinical contexts, such as primary care, hospital-based settings, and long-term care facilities.

### 2.4 Stakeholders

The Delphi study will actively recruit a geographically diverse panel of stakeholders, ensuring representation from North America, Europe, Asia, Africa, and Latin America to reflect global variations in osteoporosis and diabetes management. Stakeholders will include researchers, healthcare professionals (HCPs), and patient/public involvement (PPI) representatives from multiple regions and healthcare systems. Special efforts will be made to include perspectives from LMICs, where osteoporosis and diabetes present unique clinical challenges. Recruitment will be conducted through major international professional societies, regional osteoporosis and diabetes organizations, and direct invitations to researchers involved in multinational clinical trials.

1. Researchers: Experts with a track record in osteoporosis and DM research, including those working in academic institutions, industry, and healthcare organizations. These individuals will have experience in designing and conducting clinical trials, systematic reviews, or guidelines for the management of osteoporosis in patients with DM.
2. HCPs: This group will encompass physicians (e.g., endocrinologists, diabetologists, geriatricians, general practitioners), pharmacists, nurses, physical therapists, and other allied health professionals with direct experience in managing osteoporosis and diabetes. Their clinical expertise will provide insights into the practical aspects of outcome measurement and patient care.
3. PPI Representatives: Patients with a history of osteoporosis and DM, as well as their caregivers, will be included as PPI representatives. This group will provide valuable input on patient-centered outcomes, treatment preferences, and quality of life measures. Their involvement ensures that the COS addresses the needs and priorities of those directly affected by these conditions. The PPI representatives will participate in all phases of the Delphi process, including reviewing and providing feedback on proposed outcomes and contributing to consensus meetings.
4. Policy and Public Health Experts: Recognizing the broader implications of managing osteoporosis in patients with DM, experts involved in national health policy-making, public health advisory roles, and guideline development will be invited. Their inclusion aims to ensure that the COS is relevant not only to clinical practice but also to health policy and resource allocation.

### 2.5 Delphi participants and recruitment strategy

The recruitment of Delphi participants will follow a two-tier approach to ensure a broad representation of expertise. Initially, invitations will be extended to members of relevant scientific societies, such as the International Osteoporosis Foundation (IOF) and the European Association for the Study of Diabetes (EASD). Known experts in the field, identified through literature searches and professional networks, will also be approached directly. These experts will represent diverse settings, including primary care, secondary care, and long-term care facilities. In addition, first or last authors of at least three peer-reviewed publications on osteoporosis and DM will be invited to participate. To further diversify the panel, participants will be asked to nominate local HCPs who have clinical expertise in osteoporosis management within the DM population.

If the initial recruitment does not yield a sufficient number of participants or fails to cover the necessary diversity of expertise (target sample size: a minimum of 50 experts across different stakeholder groups), an expanded recruitment strategy will be employed. This will involve disseminating invitations through professional registration bodies, newsletters of national healthcare services, and relevant professional organizations at the regional and international levels. The recruitment will specifically target professionals involved in the care of osteoporosis in DM, including those in community health services, hospital departments, nursing homes, and geriatric assessment units.

Through this comprehensive recruitment strategy, the Delphi study aims to capture the diverse perspectives of stakeholders involved in osteoporosis management within the diabetic population. This will ensure that the finalized COS encompasses outcomes that are not only clinically relevant but also meaningful to patients and their caregivers.

### 2.6 Sampling

In this phase, we will implement a purposive sampling strategy to recruit a total of 200 participants, ensuring a balanced representation across three primary settings: hospital, community, and residential care environments. The purposive sampling approach is aimed at securing diverse expertise, which includes researchers and HCPs in equal proportions—a 50/50 ratio—to represent both academic insights and clinical practice. Additionally, we will strive for a balanced representation by gender and geographical diversity, accounting for participants’ country of professional practice. This deliberate balance is intended to ensure that the perspectives of diverse stakeholders are adequately reflected throughout the Delphi process. Given the iterative nature of the Delphi rounds and the potential for participant dropout, we aim to recruit 200 participants to secure at least 50 complete responses per setting by the conclusion of all Delphi rounds. This comprehensive sampling approach is essential to uphold the credibility and inclusiveness of the consensus process (Visser et al., 2023).

### 2.7 Patient and public involvement (PPI)

PPI will be a cornerstone of this study, ensuring that the lived experiences and preferences of individuals affected by osteoporosis and DM are incorporated throughout the entire process. PPI representatives have already contributed to the initial design and protocol development, providing feedback on the relevance and clarity of proposed outcomes. Their input has been instrumental in identifying patient-reported outcomes (PROs) that reflect real-world priorities, such as pain, functional mobility, independence, treatment burden, and overall quality of life.

To ensure meaningful PPI integration, representatives will be actively engaged throughout all study phases. PPI representatives will be recruited through established patient advocacy groups, osteoporosis and diabetes patient support networks, and healthcare institutions with existing PPI frameworks. Selection criteria include lived experience with both osteoporosis and DM, ability to articulate perspectives on treatment outcomes, and willingness to participate in the Delphi rounds and final consensus meeting. To promote diversity, we will recruit individuals from various geographical regions, socioeconomic backgrounds, and healthcare settings.

During the modified Delphi process, PPI representatives will review and rate outcomes alongside clinical experts, ensuring that patient-centered priorities are explicitly recognized. Their responses will be analyzed separately to highlight any differences in perspectives between patients and healthcare professionals. Special emphasis will be placed on PROs, which will receive dedicated discussion during the consensus meeting to ensure that they are meaningfully incorporated into the final COS.

PPI representatives will also play an active role in the consensus meeting, participating as equal stakeholders alongside healthcare professionals and researchers. Their input will be weighted equally in decision-making, with equal voting rights to ensure that patient perspectives are not overshadowed by expert opinions. Additionally, to enhance inclusivity and capture nuanced patient perspectives, qualitative feedback from PPI representatives will be gathered post-meeting. This feedback will inform the final refinement of the COS before its formalization.

PPI members have already contributed to the early stages of the study by reviewing the initial protocol and providing input on outcome selection. Initial feedback has been gathered from PPI members based in different regions, offering insights into the relevance and clarity of the Delphi survey outcomes. This engagement has been crucial in shaping a patient-centered approach that accounts for lived experiences.

To further embed PPI in our methodology, representatives will be continuously involved throughout the study. They will be asked to review and provide feedback on the draft COS after the completion of all Delphi rounds and will be invited to participate in Steering Group meetings. Their involvement in these stages will ensure that the outcomes prioritized in the COS reflect patient needs and real-world applicability. By implementing these measures, this study aims to bridge the gap between clinical expertise and patient experience, ensuring a robust, inclusive, and patient-centered final COS.

### 2.8 Data management and confidentiality

Data management practices for this Delphi study will prioritize participant confidentiality and adherence to ethical standards. Information about the study and invitations to participate will be sent to publicly available email addresses of potential experts and healthcare professionals. Participants who consent to join the study will have their email addresses stored temporarily alongside their responses to facilitate communication for subsequent Delphi rounds and to monitor attrition rates.

To ensure confidentiality, only the Core Group conducting the Delphi survey will have access to the complete list of participants. Upon completion of data collection, each participant will be assigned a unique identifier to anonymize their responses. All data collected during the study will be securely stored in a password-protected university data server, ensuring that only authorized personnel have access. Ethical approval for the conduct of the Delphi survey and all related activities has been obtained from Ethics Committee of the First Affiliated Hospital of Fujian Medical University (approval number: IEC-FOM-013-3.0).

### 2.9 Delphi rounds and consensus procedure

The Delphi study will be conducted over two rounds, each designed to gather consensus on the key outcomes for pharmacological interventions in osteoporosis among patients with diabetes mellitus. Each round will last 3 weeks, providing participants with ample time to complete the survey and minimizing the risk of incomplete data. To further optimize participation rates, reminder emails will be sent on day 14 of each round to encourage survey completion among those who have not yet responded. If necessary, additional measures such as extending the survey deadline or issuing personalized reminders will be employed to enhance response rates and ensure robust data collection.

Following the completion of each round, there will be a 4-week interval to allow sufficient time for comprehensive data analysis and preparation for the subsequent round. This interval ensures that results from the first round are thoroughly examined, enabling the Steering Group to refine the list of outcomes based on participants’ feedback and ratings. By maintaining this structured and time-bound approach, we aim to minimize participant attrition and maintain engagement throughout the Delphi process. The use of this rigorous and methodologically sound approach has been validated in other core outcome set development studies and is consistent with best practices for consensus-building in medical research.

#### 2.9.1 Round 1: rating and refining outcomes

In Round 1, participants will be asked to rate the importance of each outcome using a 1–9 Likert scale, where 1–3 represents ‘not important,’ 4–6 indicates ‘important but not critical,’ and 7–9 denotes ‘critical’ for inclusion in the COS (Williamson et al., 2012). The outcomes presented will be those identified through an extensive systematic review, as well as additional outcomes pre-selected and grouped by the Steering Group. This pre-selection process ensures that the outcomes list is comprehensive and relevant, yet manageable for participants to review.

Each outcome will be accompanied by a detailed description to ensure clarity and consistency in interpretation. Participants will be encouraged to provide justifications for their ratings in a dedicated text box and suggest any additional outcomes they consider crucial but that may have been omitted. This process allows for the inclusion of a wide range of perspectives and ensures that the COS reflects outcomes that are both scientifically valid and clinically meaningful. The involvement of a diverse panel of experts and stakeholders enhances the robustness of the results and increases the likelihood of identifying outcomes that are relevant across different healthcare settings and patient populations.

Patient-reported outcomes (PROs) will receive particular attention in Round 1, with a specific focus on outcomes identified as priorities by PPI representatives. These outcomes will include pain management, functional mobility, treatment burden, and quality of life, which are critical for patients managing both osteoporosis and DM. The Steering Group, in collaboration with PPI representatives, will ensure that validated PRO measures are included and that the final COS encompasses outcomes that genuinely matter to patients.

Recognizing that PROs are inherently subjective and may be more difficult to standardize, we have implemented several methodological strategies to enhance consensus-building and ensure robust evaluation.

1. Systematic Identification of PROs: PROs will be identified through a comprehensive systematic review, prioritizing those with validated measurement tools and broad applicability across different clinical settings.
2. Active Stakeholder Engagement: PPI representatives will be actively involved in all phases of the Delphi process to ensure that PROs reflect real-world patient concerns and lived experiences.
3. Modified Delphi Consensus Criteria: Given the potential variability in PRO ratings, a modified consensus threshold will be employed. PROs will be considered for inclusion if at least 60% of participants rate them as ‘critical’ (compared to the 75% threshold for clinical outcomes). This adjustment acknowledges the subjectivity of PROs while ensuring they receive due consideration.
4. COSMIN-Based Assessment of PROs: The measurement properties of PROs will be evaluated using the COnsensus-based Standards for the selection of health Measurement INstruments (COSMIN) criteria, ensuring that selected measures meet high standards of validity, reliability, and responsiveness.
5. Expert Review and Final Consensus Meeting: A dedicated discussion on PROs will take place during the final consensus meeting, allowing clinical experts and patient representatives to refine and validate their inclusion.

#### 2.9.2 Round 2: achieving consensus

In Round 2, the list of outcomes will be refined based on the ratings and feedback from Round 1. Outcomes that received consensus in the first round, defined as being rated as ‘critical’ by at least 75% of participants and ‘not important’ by fewer than 15%, will be retained as potential core outcomes. Conversely, outcomes rated as ‘not important’ by 75% of participants and ‘critical’ by fewer than 15% will be considered for exclusion from the COS.

For PROs, we will employ less stringent consensus criteria, recognizing the importance of patient-centered outcomes in this context. PROs will be included if over 60% of participants rate them as ‘critical’ and fewer than 15% rate them as ‘not important,’ and will be excluded if the inverse is true. This approach ensures that PROs, which might be more variable due to their subjective nature, are given due consideration.

Participants will receive individualized feedback from Round 1, including their own scores and the overall distribution of ratings across the participant group. This feedback process enhances the transparency of the Delphi method and enables participants to reconsider their initial ratings in light of the broader group consensus. Outcomes that did not reach consensus in Round 1, as well as any new outcomes proposed by participants, will be re-rated in Round 2.

### 2.10 Data management and attrition assessment

To manage data effectively, we will use a secure online survey platform that prevents accidental skipping of questions, thereby minimizing the occurrence of missing data. All participant responses will be anonymized post-collection, and data will be stored on a password-protected university server in compliance with data protection regulations. This approach is consistent with ethical research standards and ensures the confidentiality of all participants.

Potential bias due to participant attrition will be systematically assessed by comparing the outcome ratings from Round 1 between those who complete both Delphi rounds and those who drop out after Round 1. This comparison will help identify any systematic differences that could influence the study results and inform subsequent data interpretation (Williamson et al., 2017). We will employ established statistical techniques, such as chi-square tests or t-tests, to analyze differences in ratings between completers and non-completers.

#### 2.10.1 Phase 3: consensus meeting

An online consensus meeting will be conducted with 10–15 carefully selected participants to finalize the COS for pharmacological interventions in osteoporosis among patients with diabetes mellitus. These participants will be chosen from those who expressed interest in further involvement and will represent a balance of stakeholder groups, including HCPs, researchers, and two PPI representatives. Selection will also consider gender, country, professional background, and healthcare setting to ensure diverse perspectives are reflected. While the online format allows for broader international participation, it may limit the depth of discussions compared to in-person meetings. Therefore, the meeting will be structured to facilitate meaningful engagement, with experienced moderators guiding discussions to ensure balanced contributions from all participants. Ahead of the meeting, detailed briefing materials summarizing the Delphi results and key discussion points will be provided to optimize efficiency and encourage informed deliberation. Breakout sessions will be incorporated to allow in-depth discussions on specific outcome domains before reconvening for group-wide consensus-building. Additionally, participants will have the opportunity to provide supplementary feedback post-meeting to capture any nuances not fully explored during live discussions. The outcomes from the Delphi study—including those that reached consensus, those that did not, and those requiring further deliberation—will be systematically reviewed, with particular attention given to PROs and perspectives. The results from PPI participants will be summarized separately to ensure that patient experiences are meaningfully integrated into the final COS. Following structured discussions, participants will vote anonymously on whether each outcome should be included in the final COS, with inclusion requiring at least 70% agreement. To further refine consensus decisions and mitigate potential limitations of the online format, a follow-up survey will be distributed after the meeting, allowing participants to reconsider and clarify their positions where necessary.

#### 2.10.2 Phase 4: evaluation of measurement methods

Once the outcomes for the COS have been established, Phase 4 will focus on determining the most appropriate methods for measuring these outcomes. The assessment methods identified during the systematic review in Phase one will be evaluated using the COnsensus-based Standards for the selection of health status Measurement INstruments (COSMIN) criteria. These criteria assess various measurement properties, including internal consistency, reliability, measurement error, content validity, structural validity, hypothesis testing, cross-cultural validity, criterion validity, and responsiveness (Mokkink et al., 2010). The Steering Group will also have the flexibility to propose additional measurement methods that may not have been identified in the systematic review, particularly those that are newly developed or have not yet been widely published.

A core group of experts will evaluate the identified measurement methods, considering not only their validity and reliability according to COSMIN criteria but also other practical factors such as feasibility, cost, and ease of implementation in diverse healthcare settings. These aspects are crucial in ensuring that the COS can be effectively applied across different clinical contexts. The preferred measurement methods will be selected during a second virtual consensus meeting, where participants will vote using the same 70% threshold applied in Phase 3.

#### 2.10.3 Phase 5: dissemination and implementation

The final phase of the project will focus on the dissemination and implementation of the COS. The Steering Group members, along with participants from various stakeholder groups, will play an active role in promoting the COS to the broader scientific and healthcare communities. This will include the publication of the final COS in peer-reviewed journals and presentations at international conferences such as those organized by the European Society for Clinical and Economic Aspects of Osteoporosis and Osteoarthritis (ESCEO) and the International Osteoporosis Foundation (IOF). In addition, efforts will be made to engage with health policymakers, patient advocacy groups, and caregiver associations to support the integration of the COS into clinical guidelines and decision-making frameworks.

## 3 Discussion

### 3.1 Expected main findings of the study

This study aims to develop a COS for pharmacological interventions in osteoporosis among patients with diabetes mellitus through a comprehensive, evidence-based process involving expert and patient input. The expected main finding of this study is the identification and consensus on a set of outcomes that will be crucial for standardizing research and clinical practices concerning osteoporosis treatment in patients with diabetes. By addressing a population with complex comorbidities, this COS will ensure that future trials and interventions focus on the most relevant outcomes, such as fracture incidence, bone mineral density, and patient-reported outcomes like quality of life and pain levels, while accounting for diabetes-specific challenges like glycemic control and cardiovascular risks.

This COS will contribute significantly to closing the gaps in current research, where outcome heterogeneity often hampers comparisons between studies and compromises the synthesis of high-quality evidence. It is expected that through this international consensus approach, the final COS will provide a framework for uniform reporting and facilitate comparisons across clinical trials and real-world studies. Moreover, the inclusion of patient perspectives, particularly through the engagement of PPI representatives, will highlight patient-centered outcomes that are often underrepresented in clinical research. As a result, the COS is expected to improve the quality and relevance of outcomes measured in this vulnerable population, enhancing the applicability of research findings to clinical practice.

### 3.2 Comparisons with similar studies

The methodology and expected outcomes of this study are comparable to previous COS development initiatives in other clinical areas, such as rheumatoid arthritis and chronic kidney disease, where consensus-based methods have proven effective in standardizing outcome reporting (Evangelidis et al., 2021; Kirkham et al., 2017). For example, the development of a COS for rheumatoid arthritis trials significantly improved the consistency and comparability of clinical outcomes, which in turn facilitated meta-analyses and guideline development. Similarly, a COS developed for chronic kidney disease provided a clear framework for assessing both clinical and patient-reported outcomes, ensuring that future trials address outcomes that matter most to patients.

Compared to these previous COS initiatives, the current study is distinguished by its focus on a population with dual chronic conditions—osteoporosis and diabetes mellitus—both of which carry unique risks and treatment challenges. While previous COS studies have largely focused on single-disease frameworks, this study acknowledges the intersection of multiple morbidities and the need for outcome measures that capture the complexity of managing osteoporosis in the context of diabetes. As such, this study is likely to contribute novel insights into how outcome sets can be tailored for populations with complex, multi-faceted healthcare needs.

### 3.3 Strengths of the study

One of the major strengths of this study is its rigorous, systematic approach to developing the COS, which follows best practice guidelines for COS development, including the use of Delphi methodology, stakeholder engagement, and consensus meetings. The inclusion of diverse stakeholder groups—clinicians, researchers, patients, and policymakers—ensures that the final COS is both scientifically valid and clinically meaningful.

Another strength of the study is its international scope, which ensures that the COS will be applicable across different healthcare systems and settings. The involvement of experts from various regions allows for the consideration of cultural, economic, and systemic differences that may influence outcome prioritization. This global perspective is particularly important in the context of osteoporosis and diabetes, where treatment strategies and healthcare resources can vary widely between countries. By developing a COS that is relevant across different settings, the study has the potential to influence global research and practice standards for managing osteoporosis in patients with diabetes.

Additionally, the application of COSMIN criteria ensures that selected measurement tools are methodologically rigorous and clinically meaningful. By systematically evaluating measurement properties such as validity, reliability, and responsiveness, we guarantee that each selected instrument provides accurate and reproducible data. This methodological approach aligns with best practices in evidence-based medicine and enhances the credibility and applicability of the COS across different clinical and research settings. Additionally, by incorporating feasibility assessments and patient perspectives, we ensure that the final measurement tools are practical for real-world implementation, further reinforcing the robustness of the study’s methodological framework.

A key strength of this study is the structured and systematic integration of patient representatives, ensuring that the final COS reflects patient priorities while maintaining methodological rigor. Unlike traditional expert-driven COS development, this study employs a dedicated approach to PPI inclusion, incorporating patient perspectives at every stage of the process. Patient-reported outcomes (PROs), such as pain management, mobility, independence, and quality of life, are prioritized alongside traditional clinical outcomes like fracture rates and bone mineral density. To ensure that PROs are not overshadowed by clinical perspectives, PPI responses are analyzed separately during the Delphi process, and PPI representatives have equal voting rights in the consensus meeting. Post-meeting qualitative feedback mechanisms further capture nuanced patient perspectives, refining the COS and enhancing its relevance. This patient-centered approach aligns with contemporary trends in healthcare research, emphasizing the importance of integrating the patient’s voice into clinical decision-making and trial design. By embedding PPI throughout the study—from protocol development to consensus meetings—the COS will reflect outcomes that are not only scientifically robust but also meaningful to those directly impacted by osteoporosis and diabetes, ultimately improving its real-world applicability and validity for both patients and healthcare professionals.

### 3.4 Limitations of the study

Despite its strengths, this study has several potential limitations. One limitation of our study is the reliance on expert consensus, which, while a recognized and robust methodology for COS development, carries the potential risk of bias if certain regions or specialties are overrepresented. To address this, we have implemented a structured recruitment strategy to ensure geographic diversity (including experts from high-, middle-, and low-income countries), multidisciplinary representation, and a balance between researchers, clinicians, and patient representatives. Additionally, we will analyze response patterns from the Delphi process across different regions and specialties to detect and account for any potential biases in outcome prioritization. These measures are designed to enhance the generalizability and applicability of the final COS.

Another limitation is the challenge of achieving consensus on PROs, given their subjective nature and variability among patients. While this is a recognized difficulty in COS development, we have implemented several measures to address this issue. These include using a systematic review to identify validated PROs, engaging patient representatives in all phases of the study, applying COSMIN criteria to assess measurement validity, and adjusting the Delphi consensus criteria to accommodate the unique challenges posed by PROs. Additionally, a final consensus meeting will allow for further refinement and validation of selected PROs to ensure they align with both patient experiences and clinical relevance. These measures enhance the likelihood that the COS will include PROs that are both meaningful and standardized for future research.

Furthermore, the consensus meeting will be conducted online rather than in person, which may impact the depth of discussions and informal exchanges that typically occur in face-to-face meetings. However, to address this limitation, we have adopted several strategies, including structured facilitation, pre-meeting briefing materials, breakout discussion groups, post-meeting written feedback, and follow-up surveys. These measures are designed to enhance interaction and ensure a robust and inclusive consensus process. Additionally, the online format increases accessibility and allows for the participation of a more diverse group of international experts and patient representatives, ultimately strengthening the global applicability of the final Core Outcome Set.

### 3.5 Importance of the study and conclusion

The development of a COS for pharmacological interventions in osteoporosis among patients with diabetes is a critical step forward in addressing a significant gap in clinical research and practice. By standardizing outcome reporting, this study will enable more meaningful comparisons across trials, improve the quality of evidence available for clinical decision-making, and ultimately enhance patient care. The focus on both clinical and patient-reported outcomes ensures that the COS will reflect a holistic approach to managing osteoporosis in patients with diabetes, aligning with the broader trend toward patient-centered care in chronic disease management.

In conclusion, this study is expected to have a significant impact on both research and clinical practice by providing a standardized set of outcomes that are relevant, reliable, and feasible for use in a variety of settings. The involvement of an international panel of experts and stakeholders, combined with a rigorous methodological approach, ensures that the final COS will be applicable globally and adaptable to diverse healthcare environments. This study will also contribute to the growing body of evidence supporting the importance of patient-reported outcomes in clinical research, reinforcing the need for interventions that address both clinical and quality-of-life outcomes in patients with complex comorbidities.

## Data Availability

The original contributions presented in the study are included in the article/supplementary material, further inquiries can be directed to the corresponding authors.
